# Optimization of quasi‐diffusion magnetic resonance imaging for quantitative accuracy and time‐efficient acquisition

**DOI:** 10.1002/mrm.29420

**Published:** 2022-08-31

**Authors:** Catherine A. Spilling, Franklyn A. Howe, Thomas R. Barrick

**Affiliations:** ^1^ Neurosciences Research Section, Molecular and Clinical Sciences Research Institute St George's University of London London United Kingdom; ^2^ Centre for Affective Disorders, Department of Psychological Medicine, Division of Academic Psychiatry Institute of Psychiatry, Psychology and Neuroscience, King's College London London United Kingdom

**Keywords:** non‐Gaussian diffusion MRI, optimization, quasi‐diffusion MRI

## Abstract

**Purpose:**

Quasi‐diffusion MRI (QDI) is a novel quantitative technique based on the continuous time random walk model of diffusion dynamics. QDI provides estimates of the diffusion coefficient,$\;D_{1,2}$ in mm^2^ s^−1^ and a fractional exponent, α, defining the non‐Gaussianity of the diffusion signal decay. Here, the *b*‐value selection for rapid clinical acquisition of QDI tensor imaging (QDTI) data is optimized.

**Methods:**

Clinically appropriate QDTI acquisitions were optimized in healthy volunteers with respect to a multi‐*b*‐value reference (MbR) dataset comprising 29 diffusion‐sensitized images arrayed between b=0 and 5000 s mm^−2^. The effects of varying maximum *b*‐value ($b_{\max}$), number of *b*‐value shells, and the effects of Rician noise were investigated.

**Results:**

QDTI measures showed $b_{\max}$ dependence, most significantly for α in white matter, which monotonically decreased with higher $b_{\max}$ leading to improved tissue contrast. Optimized 2 *b*‐value shell acquisitions showed small systematic differences in QDTI measures relative to MbR values, with overestimation of$\;\;D_{1,2}$ and underestimation of α in white matter, and overestimation of $D_{1,2}$ and α anisotropies in gray and white matter. Additional shells improved the accuracy, precision, and reliability of QDTI estimates with 3 and 4 shells at $b_{\max}=5000$ s mm^−2^, and 4 *b*‐value shells at $b_{\max}=3960$ s mm^−2^, providing minimal bias in $D_{1,2}$ and α compared to the MbR.

**Conclusion:**

A highly detailed optimization of non‐Gaussian dMRI for in vivo brain imaging was performed. QDI provided robust parameterization of non‐Gaussian diffusion signal decay in clinically feasible imaging times with high reliability, accuracy, and precision of QDTI measures.

## Abstract 2

Click here for author‐reader discussions

## 1 INTRODUCTION

Diffusion‐weighted magnetic resonance imaging (dMRI) is sensitive to the effects of random translational motion of water molecules at the length scale of cellular and subcellular structures, allowing interrogation of healthy and pathological tissue properties beyond nominal image resolution. Quasi‐diffusion magnetic resonance imaging (QDI), a novel non‐Gaussian dMRI signal attenuation modeling technique that provides rapid acquisition, was recently proposed.^1^ QDI provides analogous tissue contrast and microstructural inference to diffusional kurtosis imaging (DKI).^2^, ^3^ QDI is mathematically robust. It provides feasible QDI parameter estimates in voxels, for which DKI assigns infeasible negative excess kurtosis, without requiring regularization^1^, ^4^ and represents the dMRI signal attenuation by a completely monotonic decreasing function unlike DKI.^5^

The continuous time random walk (CTRW) model of diffusion^6^ provides a mathematical description of dMRI signal decay with a physical interpretation of diffusion dynamics, which makes no assumptions about tissue geometry. The spin's random walk is described by 2 discrete stochastic processes, with fractional exponents relating to step lengths, β, over time intervals α. Quasi‐diffusion is a special case of the CTRW model, which assumes a Gaussian scaling relationship between space and time fractional exponents, β=2α, which leads to mean squared displacement that is linearly proportional to diffusion time. In QDI, the dMRI signal decay is defined by a stretched Mittag‐Leffler function parameterized by the rate of decay (diffusion coefficient $D_{1,2}$ in mm^2^ mm^2^ s^−1^) and the shape of the power law tail (the fractional exponent, α).^1^ QDI is a model‐based quantitative alternative to DKI that indicates non‐Gaussian diffusion for 0<α<1, and Gaussian diffusion when α=1 . 1, 5

The quasi‐diffusion model has similar assumptions to the random permeable barriers model^7^, ^8^ — it assumes local Gaussian diffusion propagators generate an ensemble average of diffusing spins, which is observed as non‐Gaussian dMRI signal attenuation within a voxel.^5^ The mathematics and interpretation of QDI enable direct derivation of a spectrum of Fickian diffusion coefficients within a voxel and a quasi‐diffusion propagator via the inverse Laplace and Fourier transforms, respectively.^5^ This mathematical formulation is currently unique among dMRI signal representation and modeling techniques — QDI is the only approach in the literature, which has closed forms for its propagator and both the Fourier and Laplace transforms. The form of the propagator also allows derivation of quasi‐diffusion mean apparent propagator (qMAP) imaging,^5^ which is a model based alternative to standard MAP imaging.^9^

The mathematical description of QDI provides insight into the heterogeneity of the diffusion environment, therefore, $D_{1,2}$ and α will be similarly sensitive to pathology as DKI and MAP techniques.^1^, ^5^ Recent evidence suggests that QDI provides comparable sensitivity to age‐related changes in white matter complexity as DKI.^10^ Consequently, QDI has potential applications in quantitative tissue microstructural research and clinical imaging studies.

A recent study showed that neuroradiologists endorse quantitative MRI (qMRI) to improve diagnostic accuracy, however, there has been limited clinical translation of qMRI into clinical neuroradiology, with DWI the most commonly used qMRI technique and DKI the least.^11^ DWI is a particularly important sequence for oncology and recommendations for improving precision of conventional DWI for calculating ADC maps have been suggested.^12^ Moreover, a recent meta‐analysis suggests DKI has higher diagnostic accuracy for cancer screening than standard DWI.^13^ QDI offers an alternative to DKI for simultaneously assessing non‐Gaussian diffusion and DWI/DTI measures.

Clinical translation of quantitative QDI requires standardization of acquisition and processing, assessment of parameter precision and accuracy, and the ability to acquire high‐quality data within clinically appropriate times.^14^ Development and assessment of qMRI techniques involve 4 types of studies^15^: (1) simulations^16^, ^17^ that allow absolute control of imaging and sample parameters, but are the least realistic; (2) phantom studies^18^, ^19^, ^20^ that include actual instrumental errors and a stable parametric ground truth, although absolute accuracy may not be known by standard assay; (3) pre‐clinical studies,^21^, ^22^, ^23^, ^24^, ^25^, ^26^ designed to validate imaging measurements against actual tissue characteristics, but where instrumental measurement errors and tissue characteristics may not be fully representative of measurements made in humans; and (4) patient^27^ and healthy volunteer studies^16^, ^20^, ^28^ that provide actual tissue characteristics and instrumental measurement errors, but have unknown ground truth parameters, which are inferred from highly detailed or repeated measurements.

Our study primarily takes the fourth approach, assessing the accuracy and precision of quasi‐diffusion tensor imaging (QDTI) measures of mean $D_{1,2}$ and α and their anisotropies, in normal brain tissue using healthy volunteers. We aim to determine optimal protocols with minimal acquisition times on a standard 3T clinical MR system. Here, the ground truth is unknown, but we assume that a dMRI acquisition with a large number of *b*‐value shells will lead to signal decay from which accurate voxel‐by‐voxel $D_{1,2}$ and α estimates can be obtained as a reference against which to compare faster, more clinically acceptable acquisitions. Similar methodology has been used for optimizing kurtosis measures from DKI in healthy volunteers^16^, ^28^ and stroke patients,^27^ as well as for optimizing microstructural parameters derived from complex multi‐dimensional models^29^, ^30^ and for optimizing novel formulations of the diffusion kurtosis tensor.^4^, ^31^, ^32^

Our definition of a “conventional clinical dMRI” is a 6‐direction single‐shot DTI protocol acquired with high in‐plane resolution and thick axial slices, typically acquired in 1 min. Our QDTI optimization is performed with a similar spatial resolution and with reference to a comprehensive dMRI acquisition acquired in 6 non‐collinear diffusion gradient directions across 29 *b*‐values equally spaced between 0 and 5000 s mm^−2^. We minimize the normalized sum of squared error (NSSE) between fitted QDI decay curves for different *b*‐value shell combinations and the QDI decay curves obtained from the multi‐*b*‐value reference (MbR) up to a defined maximum *b*‐value (*b*_max_). Optimal QDTI protocols with 6 diffusion gradient directions are identified for $b_{\max}$ of 2000, 3000, 4000, and 5000 s mm^−2^ (therefore, enabling representative results for scanners with lower gradient strengths than our maximum of 80 mT m^−1^) and reliability, accuracy, and precision relative to the MbR value, are investigated. Furthermore, we investigate the behavior of $D_{1,2}$ and α parameters as $b_{\max}$ is increased and describe the effects on QDTI measures calculated for $2000\leq b_{\max}\leq 5000\;$s mm^−2^.

Effects of Rician noise in magnitude images are dependent on SNR^17^ and maximum *b*‐value in DKI^16^ for which tissue contrast is dependent on $b_{\max}$.^16^ These effects can lead to significant overestimation of kurtosis in DKI measurements^33^ and affect complex metrics derived from high *b*‐value multi‐shell dMRI data.^34^ Here, we use a model‐based approach to investigate the effects of noise on QDTI measures. Various methods have been devised to compensate for these noise effects, from simple bias correction assuming Rician noise^33^ to model‐free noise mapping and signal correction.^35^ The more complex approaches are needed for multi‐coil reconstructions where image noise characteristics are spatially variant with more complex distributions than Rician.^36^, ^37^ In this study, we process diffusion data without noise reduction and use image noise levels estimated from our data to model its effects on QDTI measures.

## 2 METHODS

### 2.1 Participants

Five young healthy volunteers (age, 22 ± 4.5 years; *n* = 5 male participants) were recruited from St. George's University of London. Ethical approval for the study was granted by East London 3 Research Ethics Committee (10/H0701/36). All individuals provided informed written consent before MRI.

### 2.2 Magnetic resonance image acquisition

MRI was acquired on a 3T Philips Achieva Dual TX MR system (Philips Healthcare, Best, Netherlands) equipped with a 32‐channel head coil. Whole‐brain axial dMRI were acquired using a single‐shot diffusion sensitized spin‐echo planar imaging sequence in enhanced gradient mode (maximum amplitude 80 mT m^−1^, slew rate 100 mT m^−1^ ms^−1^) in 6 diffusion gradient directions equally spaced on the hemisphere (TE = 90 ms, TR = 6000 ms, FOV = 210 × 210 mm with 22 5‐mm thick slices acquired at 2.3 × 2.3 × 5 mm resolution and zero‐filled to provide 1.5 × 1.5 × 5 mm). Fat suppression was achieved using spectral presaturation inversion recovery (SPIR) and slice selection gradient reversal (SSGR). A SENSE factor 2 and half scan factor 0.891 was used to minimize echo‐train length and acquisition time.

DWIs were acquired in 4 blocks to minimize the effects of subject movement and scanner drift. Each block included 4 images without diffusion sensitization (b=0 s mm^−2^) followed by interleaved *b*‐values from b=180 to 5000 s mm^−2^ (δ=23.5 ms, ∆=43.9ms) with number of shot averages (NSA)=2 to increase SNR. Diffusion weightings for each block were: b={0,180,900,1620,2340,3060,3780,4500,5000} s mm^−2^, b={0,360,1080,1800,2520,3240,3960,4680,5000} s mm^−2^; b={0,540,1260,1980,2700,3420,4140,4860,5000} s mm^−2^; and b={0,720,1440,2160,2880,3600,4320,5000} s mm^−2^. This provided an acquisition with 28 *b*‐value shells at intervals of 180 s mm^−2^ with total acquisition time of 40 min 24 s. Sagittal 3D T_1_‐weighted volume images were acquired using a turbo field echo sequence (TE=3.73ms, TR=7.8ms, flip angle=8°, FOV=240×240mm, 140 slices of thickness 1.5 mm) providing voxel resolution of 1×1×1.5mm within an acquisition time of 6 min 19 s.

### 2.3 Image analysis

#### 2.3.1 Diffusion‐weighted image pre‐processing

Simultaneous eddy current and movement correction was performed for each dMR image to an average b=0 s mm^−2^ image using FSL (version 5.0.11, https://fsl.fmrib.ox.ac.uk/fsl/fslwiki/).^38^ Data were skull stripped using FSL and the normalized signal attenuation ($S_{b}/S_{0}$) computed across all *b*‐value shells. No noise reduction or spatial smoothing were performed. Whole brain gray and white matter tissue regions of interest (ROIs) were defined on QDTI maps via coregistration of high‐probability (95% likelihood) T_1_‐weighted tissue segmentations (see Supporting Information section S1).

#### 2.3.2 Quasi‐diffusion tensor imaging

The quasi‐diffusion model is described by the Mittag‐Leffler function (MLF),

(1) $$E_{\alpha }(z)=\sum_{k=0}^{\infty }\frac{z^{k}}{\Gamma (\alpha k+1)}.$$

For 0<α≤1, where Γ(x) is the gamma function. The MLF can be considered to be a generalization of the exponential function and is completely monotone in the negative real axis for 0<α≤1.^5^ The quasi‐diffusion signal attenuation, S(b), at a given diffusion‐sensitization, b, (in s mm^−2^) is given by,

(2) $$\frac{S(b)}{S(0)}=E_{\alpha }\left(-{\left(bD_{1,2}\right)}^{\alpha }\right)\;,$$

where $D_{1,2}$ is the diffusion coefficient in mm^2^ s^−1^, and α is the fractional exponent. Equation (2) describes Gaussian diffusion when α=1 and non‐Gaussian diffusion for 0<α<1. The fractional exponent is indicative of the inverse power law of the diffusion signal attenuation.^1^, ^5^ $D_{1,2}$, and α, are estimated by fitting Equation (2) to acquired dMRI data.^1^ In this study, quasi‐diffusion model fitting was performed in each diffusion gradient direction to a minimum of 3 *b*‐values on a voxel‐by‐voxel basis using a Levenberg–Marquardt algorithm (http://www.gnu.org/software/gsl). Data fitting was initialized in each voxel using expected values for Gaussian diffusion with $D_{1,2}=2.98\times 10^{-3}$ mm^2^ s^−1^ and α=0.978 providing robust initial fitting conditions. Padé approximation was used to rapidly estimate the MLF and its derivatives.^39^ The fitting procedure estimates $D_{1,2}$ and α along each diffusion gradient direction. Supporting Information Figure S1 shows QDI model fits to MbR data with $b_{\max}=5000$ s mm^−2^ for representative gray and white matter voxels.

Parameter estimates in each diffusion gradient direction are considered to be spherical samples, from which 3×3 symmetric tensors of $D_{1,2}$ and α may be calculated within a voxel.^1^, ^5^, ^40^ Here, we use a novel approach to calculate the α tensor, A, that better uses the directional residuals of ${\left(D_{1,2}\right)}^{\alpha }$. Along a given diffusion gradient direction, $g=\left(g_{x},\;g_{y},\;g_{z}\right)$, we have

(3) $$y_{g}={\left(D_{1,2}\right)}_{g}^{\alpha_{g}},$$

and

(4) $$\alpha_{g}=\frac{\ln\left(y_{g}\right)}{\ln{\left(D_{1,2}\right)}_{g}}.$$

To allow general linear model estimation of the α tensor, A, we use the matrix logarithm to obtain an equation for A using the $D_{1,2}$ tensor as,

(5) $$g^{T}\mathrm{Ag}=\frac{\ln{\left(D_{1,2}\right)}_{g}^{\alpha_{g}}}{g^{T}Q\ln\left(\Lambda \right)Q^{T}g},$$

where Q is the eigenvector matrix of the $D_{1,2}$ tensor and Λ is its matrix of eigenvalues. Each tensor was diagonalized and mean and fractional anisotropy maps were calculated from the $D_{1,2}$ and tensors,^1^ along with axial and radial maps. Here, we consider α anisotropy to describe the anisotropy of the heterogeneity of the tissue microenvironment.

#### 2.3.3 Investigation of the effect of maximum *b*‐value on QDTI measures

Before optimization for minimum acquisition time, we investigated whether the behavior of QDTI measures in whole brain gray and white matter ROIs is affected by $b_{\max}$ using subsets of the MbR data, in which $b_{\max}$ was increased from 1980 to 5000 s mm^−2^. QDTI maps were computed from each MbR subset from 1980 to 5000 s mm^−2^, giving 18 sets of QDTI data. Mean, axial, radial, and anisotropy measures for $D_{1,2}$ and α were calculated within gray and white matter ROIs for each MbR subset.

#### 2.3.4 Investigation of the effect of Rician noise on quasi‐diffusion model fitting

Full methods for investigation of Rician noise in QDI can be found in the Supporting Information section S2. In brief, Rician noise levels were estimated from ventricular CSF based on the assumption of a Rayleigh noise distribution. Noise‐free decay curves were calculated for CSF, cortical gray matter and callosal white matter for mean $D_{1,2}$ and α values obtained by fitting the quasi‐diffusion model to all acquired *b*‐values. Simulated Rician noise (based on the standard deviation of estimated Gaussian noise, σ) was added at each *b*‐value within our simulated dMRI signal (1000 simulations at different noise levels). Means and standard deviations of estimated $D_{1,2}$ and α values were calculated from MLF fits to the simulated noisy decay curves.

#### 2.3.5 Optimization of *b*‐values for quasi‐diffusion model fitting

Optimization was performed to match the shape of the fitted decay curves by minimizing the NSSE along all 6 diffusion gradient directions rather than optimizing for $\;D_{1,2}$ and α separately or minimizing a cost function that included magnitudes or weightings of $D_{1,2}$ and α estimates. This strategy ensured the existence of a single solution to the optimization problem and simultaneously provided optimization of QDTI maps. Several optimization experiments were performed to obtain minimal data acquisition protocols while maximizing image quality, precision, and accuracy for different $b_{\max}$:

1. **Two** ***b*** **‐value shells.** To derive acquisition protocols for 3T scanners across a range of gradient strengths, we optimized for fixed $b_{\max}$ of approximately: 2000, 3000, 4000, and 5000 s mm^−2^ (actual MbR *b*‐values: 1980, 3060, 3960, and 5000 s mm^−2^). The most rapidly acquired optimal solution for each $b_{\max}$ will include the b=0 s mm^−2^ data and 2 *b*‐value shells (acquisition time 3 min 12 s). The voxelwise NSSE between the fitted curves for the MbR (the expected measure computed across the *b*‐value range $0\leq b\leq b_{\max}$) and that from each permutation of 2 *b*‐values (a subset of the MbR data) was calculated in each diffusion gradient direction. NSSE was computed across evenly spaced *b*‐values (step size of b=100 s mm^−2^) from 0 to $b\leq b_{\max}$. The NSSE was averaged across gradient directions to provide a single error value at each voxel for each subject. *b*‐value optimization was performed by identifying the *b*‐value combination with the minimum cohort average (n=5) of the median error within the brain tissue ROI.
2. **Investigation of the effect of including up to 4** ***b***‐**value shells.** Further analysis was performed to investigate whether QDI parameter estimates were improved by using 3 and 4 *b*‐value shells.

#### 2.3.6 Assessment of image contrast and quality

The tissue contrast parameter, $t_{c}^{1}$ provides a measure of global image contrast between gray and white matter. Higher *t*_*c*_ indicates greater separation of gray and white matter measures, and greater visual tissue delineation when there is less variability in estimated tissue values across the brain because of reduced noise. *t*_c_ was used to compare visual quality of QDTI maps obtained from the MbR and optimal combinations for 2, 3, and 4 *b*‐value shells.

### 2.4 Statistical analysis

QDTI measures (including mean and anisotropy of $D_{1,2}$ and α) were calculated from the optimized QDI acquisitions and compared to MbR values for each $b_{\max}$. Intraclass correlation coefficients (ICC) were used to test the reliability of voxelwise measures computed from the optimal *b*‐value datasets relative to the MbR. ICC values were defined as excellent (ICC>0.9), good (0.75<ICC≤0.9), moderate (0.5<ICC≤0.75) or poor (ICC≤0.5).^41^ Relationships were visualized using scatter and Bland Altman plots.^42^ Average and standard deviation of cohort QDTI measures are reported in gray and white matter ROIs. Accuracy (difference between means) and precision (standard deviation of voxelwise differences) of the QDTI measures in comparison to the MbR are also reported in gray and white matter ROIs.

## 3 RESULTS

### 3.1 Effect of maximum *b*‐value on QDTI parameters

**FIGURE 1:**
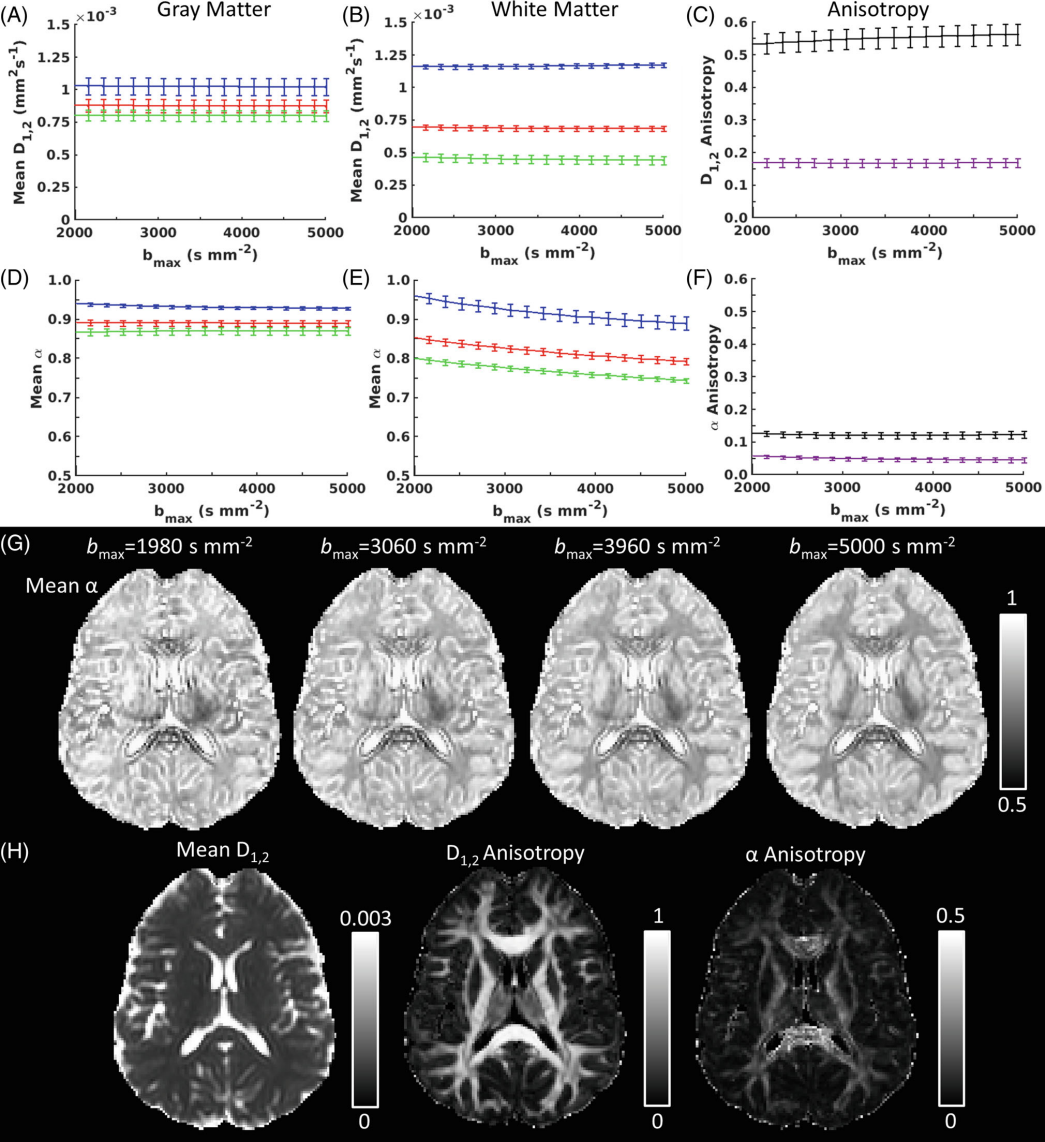
The effect of *b*‐value range on quasi‐diffusion tensor (QDTI) measures. Graphs of QDTI measures against $b_{\max}$ (over the range 2000≤b≤5000 s mm^−2^) are shown for: (A) axial, mean, and radial $D_{1,2}$ in gray matter, (B) axial, mean, and radial $D_{1,2}$ in white matter, (C) $D_{1,2}$ anisotropy, (D) axial, mean, and radial α in gray matter, (E) axial, mean, and radial α in white matter, and (F) α anisotropy. Cohort means ± standard deviations are presented. Axial = blue, mean = red, radial = green, gray matter anisotropy = magenta, white matter anisotropy = black. Row (G) shows mean α maps for $b_{\max}$ of 1980, 3060, 3960, and 5000 s mm^−2^. Row (H) shows mean $D_{1,2}$, $D_{1,2}$ anisotropy, and α anisotropy maps for a $b_{\max}$ of 5000 s mm^−2^.

Figure 1 shows the $b_{\max}$ dependence of QDTI measures from MbR data. Statistical significance of trends (Table 1) was determined from paired *t* tests between measures at $b_{\max}$ 1980 and 5000 s mm^−2^. Bonferroni corrected *P*‐values indicated significant reductions with $b_{\max}$ for gray matter mean (−0.6%), axial (−0.8%), and radial (−0.5%) $D_{1,2}$ and no change in $D_{1,2}$ anisotropy. White matter showed a significantly decreased mean (−1.9%) and radial (−5.3%) $D_{1,2}$, but an increased axial $D_{1,2}$ (0.9%), leading to significantly increased anisotropy (5.6%) with $b_{\max}$. After multiplicity correction there was a significant decrease in axial α (−1.4%) with $b_{\max}$ for gray matter, and a concomitant significant decrease in α anisotropy (−22%), this latter being a large percentage change of an absolute value close to 0. White matter showed significant reductions in α with $b_{\max}$ for mean (−7.1%), axial (−7.4%), and radial (−7.1%) measures and no resultant change in anisotropy.

**TABLE 1:** QDTI measures computed over different *b*‐value ranges in the multi‐*b*‐value reference

		MbR *b*‐value range (s mm^−2^)				
	Tissue type	0≤b≤1980	0≤b≤3060	0≤b≤3960	0≤b≤5000	Paired *t* test (*T*, *P*)
$D_{1,2}$(×10^−3^ mm^2^ s^−1^)						
Mean	Gray	0.880 ± 0.049	0.878 ± 0.049	0.877 ± 0.049	0.875 ± 0.049	−15.81^a^, <0.001^a^
	White	0.700 ± 0.020	0.691 ± 0.020	0.687 ± 0.020	0.687 ± 0.021	−14.88^a^, <0.001^a^
Axial	Gray	1.031 ± 0.065	1.027 ± 0.065	1.025 ± 0.065	1.023 ± 0.065	−10.33^a^, <0.001^a^
	White	1.164 ± 0.017	1.164 ± 0.017	1.168 ± 0.017	1.175 ± 0.016	−14.70^a^, <0.001^a^
Radial	Gray	0.805 ± 0.041	0.804 ± 0.041	0.803 ± 0.042	0.801 ± 0.042	−7.30^a^, 0.002^a^
	White	0.468 ± 0.031	0.453 ± 0.031	0.447 ± 0.032	0.443 ± 0.032	−27.95^a^, <0.001^a^
Anisotropy	Gray	0.170 ± 0.014	0.168 ± 0.013	0.168 ± 0.013	0.169 ± 0.133	−2.65, 0.057
	White	0.533 ± 0.030	0.548 ± 0.031	0.557 ± 0.032	0.563 ± 0.032	30.21^a^, <0.001^a^
Fractional exponent, α						
Mean	Gray	0.892 ± 0.007	0.891 ± 0.007	0.890 ± 0.007	0.890 ± 0.008	−5.25, 0.063
	White	0.854 ± 0.008	0.825 ± 0.009	0.808 ± 0.009	0.793 ± 0.009	−44.08^a^, <0.001^a^
Axial	Gray	0.941 ± 0.004	0.932 ± 0.004	0.930 ± 0.004	0.928 ± 0.004	−9.33^a^, <0.001^a^
	White	0.961 ± 0.014	0.924 ± 0.015	0.905 ± 0.016	0.890 ± 0.017	−24.83^a^, <0.001^a^
Radial	Gray	0.867 ± 0.009	0.870 ± 0.009	0.870 ± 0.009	0.870 ± 0.010	2.30, 0.083
	White	0.801 ± 0.007	0.775 ± 0.007	0.759 ± 0.006	0.744 ± 0.006	−46.45^a^, <0.001^a^
Anisotropy	Gray	0.059 ± 0.005	0.050 ± 0.006	0.047 ± 0.006	0.046 ± 0.008	−6.65^a^, 0.003^a^
	White	0.127 ± 0.008	0.121 ± 0.009	0.121 ± 0.009	0.124 ± 0.011	−1.46, 0.217

*Notes*: Cohort averages ± standard deviations across subjects are reported within whole brain gray and white matter regions of interest. Paired *t* tests were performed comparing QDTI measures calculated from the multi‐*b*‐value reference (MbR) data for the ranges 0 ≤ *b* ≤ 5000 compared to 0 ≤ *b* ≤ 1980. *t*‐statistics (T) and *P*‐values (P) are reported.

^a^ Significant results after Bonferroni correction for 16 measures (*P* < 0.0031).

### 3.2 Effects of Rician noise on quasi‐diffusion model fitting

**FIGURE 2:**
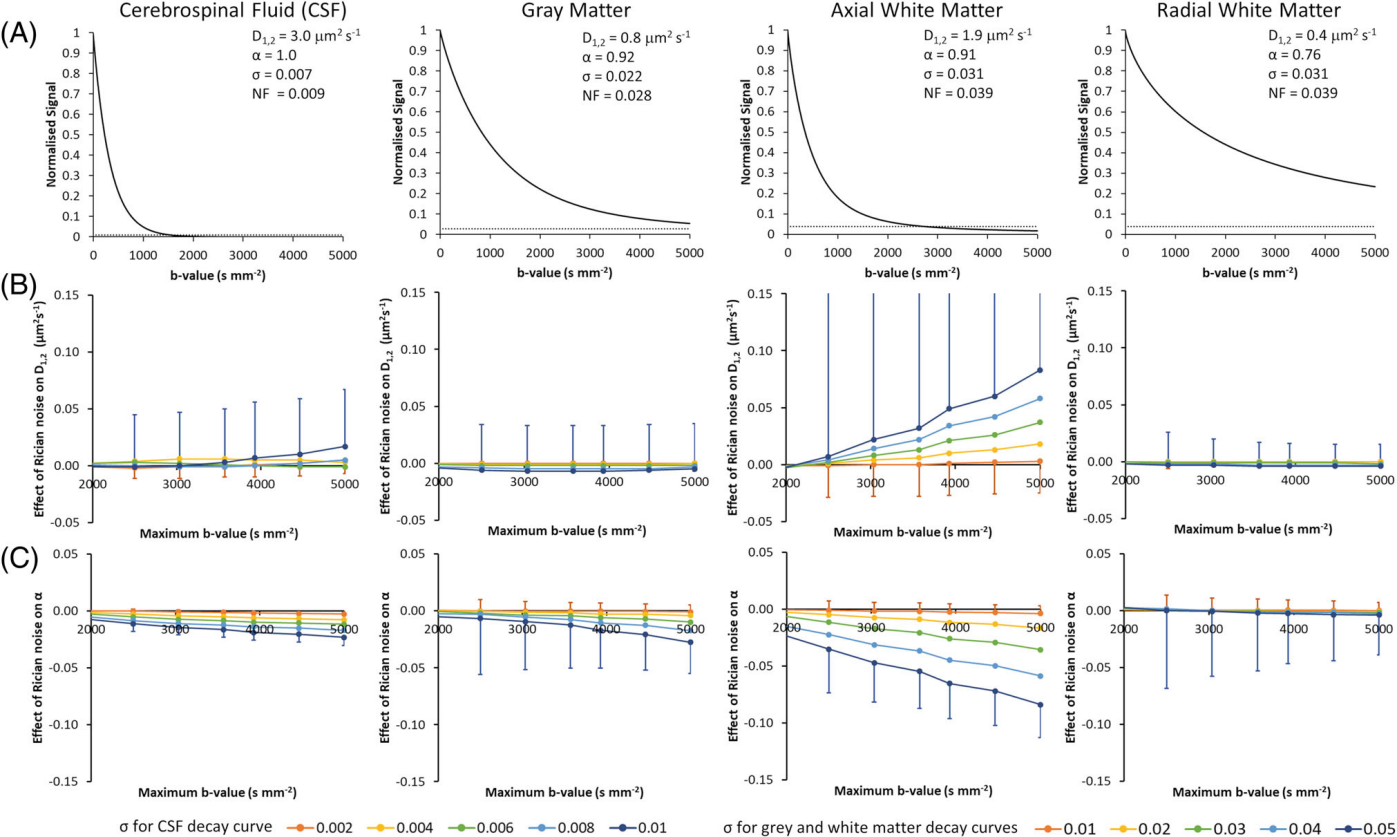
The effect of Rician noise on the estimation of $D_{1,2}$ and α for our comprehensive *b*‐value acquisition. Row (A) shows “noise free” quasi‐diffusion signal decay curves calculated from $D_{1,2}$ and α values (from left to right) CSF, gray matter, axial white matter, and radial white matter. The Rician noise floor (NF) is shown for each tissue type by the horizontal dotted line. Rows (B) and (C) show graphs of differences between $D_{1,2}$ and α estimates from simulated noisy data compared to the “noise free” values (“noise free” minus noisy estimates) across a range of σ noise levels. The simulation used the same set of *b*‐values as our dMRI acquisition. The x‐axis indicates the maximum *b*‐value over which the quasi‐diffusion model fitting was performed. Noise simulation results are shown over the range $1980\leq b_{\max}\leq 5000\;$s mm^−2^ at $b_{\max}$ values closest to intervals of 500 s mm^−2^. Circular symbols represent the mean of 1000 noise simulations and capped lines indicate the standard deviation. For clarity, the standard deviations are only shown 1‐sided and only for the maximum and minimum levels of noise used in the simulations.

Figure 2B,C show that effects of Rician noise on the estimation of $D_{1,2}$ and α are dependent on the diffusion characteristics of the tissue. Further results are presented in the Supporting Information section S3. The smallest effect is for white matter radial measurements, for which the dMRI signal does not fall below the typical noise floor (NF), and on average, for any $b_{\max}$ and noise σ there is no net shift in either $D_{1,2}$ or α. CSF rapidly decays to below the NF by $b_{\max}\approx 1500$ s mm^−2^ and for gray matter it is just above the NF at $b_{\max}\approx 5000$ s mm^−2^. This results in a negligible effect of noise on the average estimate of $D_{1,2}$, but a systematic underestimation of α that increases with increased noise and increasing $b_{\max}$. For the noise levels estimated from the image center, there is an α shift of −0.15 (−15%) for CSF and −0.04 (−5%) for gray matter.

In practice, for cortical gray matter (that is closer to the RF coil components and has higher SNR) there would be a negligible shift. The greatest effects of Rician noise are for axial white matter measurements, for which the dMRI decay curve will fall below the typical NF in central regions of the image by $b_{\max}\approx 2500$ s mm^−2^, leading to systematic increases in $D_{1,2}$ and decreases in α with increased noise and increased $b_{\max}$. For white matter close to the central coil region, we estimate axial $D_{1,2}$ could be overestimated by 0.04 mm^2^ s^−1^ (2%) on average, and axial α underestimated by 0.04 (4%) on average.

### 3.3 Optimization of QDI for reduced data acquisition times

#### 3.3.1 Minimization of NSSE to obtain optimal *b*‐value combinations

**FIGURE 3:**
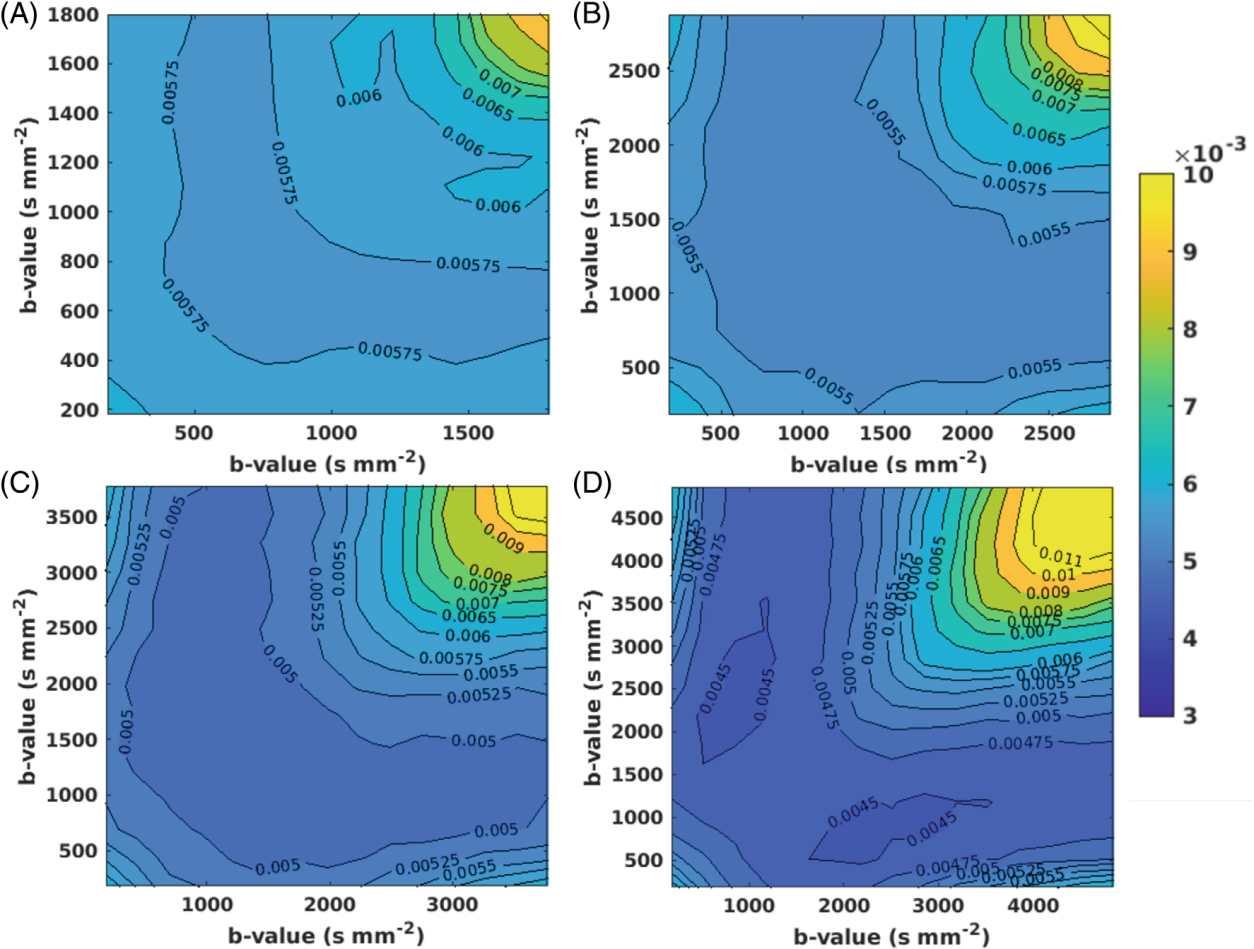
Contour maps showing cohort average normalized sum of squared error (NSSE) surfaces for 3 *b*‐value shell combinations with $b_{\max}$ of (A) 1980, (B) 3060, (C) 3960, and (D) 5000 s mm^−2^. A broad range of *b*‐value combinations produced QDI fitted decay curves, which closely matched those fitted to the multi‐*b*‐value reference (MbR) i.e. low NSSE (dark blue).

NSSE minima were used to indicate *b*‐value combinations that best matched the MbR decay curves across each diffusion gradient direction for $b_{\max}$ of 1980, 3060, 3960, and 5000 s mm^−2^ and for 2, 3, and 4 *b*‐value shells. Low NSSE values were found across a range of *b*‐value combinations for the 2, 3, and 4 *b*‐value shell analyses. Figure 3 illustrates this using contour plots for the 3 *b*‐value shell analysis for each $b_{\max}$ and demonstrates a broad floor of possible low NSSE acquisition protocols. Optimal *b*‐value shell combinations were as follows:

- 2 *b*‐value shells: b={0,540,1980}, b={0, 900, 3060}, b={0, 1080, 3960} and b={0, 1080, 5000}s mm^−2^.
- 3 *b*‐value shells: b={0, 540, 900, 1980}, b={0, 900, 1440, 3060}, b={0, 1080, 2160, 3960} and b={0, 540, 2160, 5000} s mm^−2^,
- 4 *b*‐value shells: b={0, 540, 900, 1440, 1980},b={0, 900,1260, 2160, 3060}, b={0, 540, 1620, 2160, 3960}, and b={0, 540, 1980, 2160, 5000} s mm^−2^.

#### 3.3.2 Reliability, accuracy and precision of QDTI measures for optimal 2 *b*‐value combinations

**FIGURE 4:**
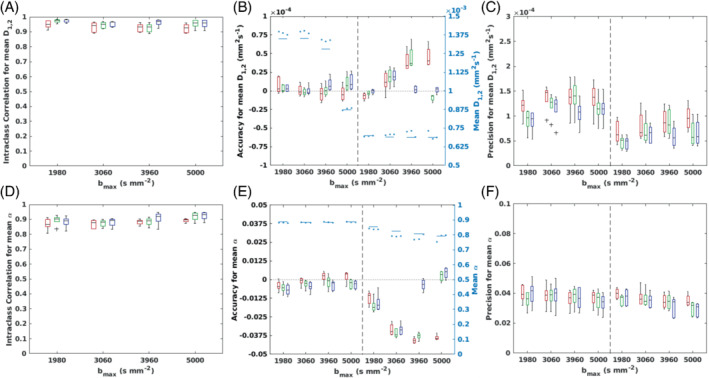
Box plots showing the reliability (intraclass correlation coefficient [ICC]), accuracy and precision (with respect to the multi‐b‐value reference [MbR]) of voxelwise quasi‐diffusion tensor (QDTI) measures within brain tissue. These are shown for optimal *b*‐value combinations with 2 (red), 3 (green), and 4 (blue) *b*‐value shells for $b_{\max}$ of 1980, 3060, 3960, and 5000 s mm^−2^. Plots are presented for mean $D_{1,2}$ showing (A) reliability, (B) accuracy, and (C) precision, and for mean α showing (D) reliability, (E) accuracy, and (F) precision. Reliability was defined as excellent (ICC > 0.9), good (0.75< ICC ≤ 0.9), moderate (0.5< ICC ≤ 0.75) or poor (ICC ≤ 0.5). Plots of ICC show results within brain tissue. Gray matter (left) and white matter (right) results are shown in the accuracy and precision plots separated by a vertical black dashed line. The second y‐axis in the accuracy plots (colored cyan) shows the mean tissue values for the MbR (solid lines) and optimal *b*‐value combinations (circular markers).

**FIGURE 5:**
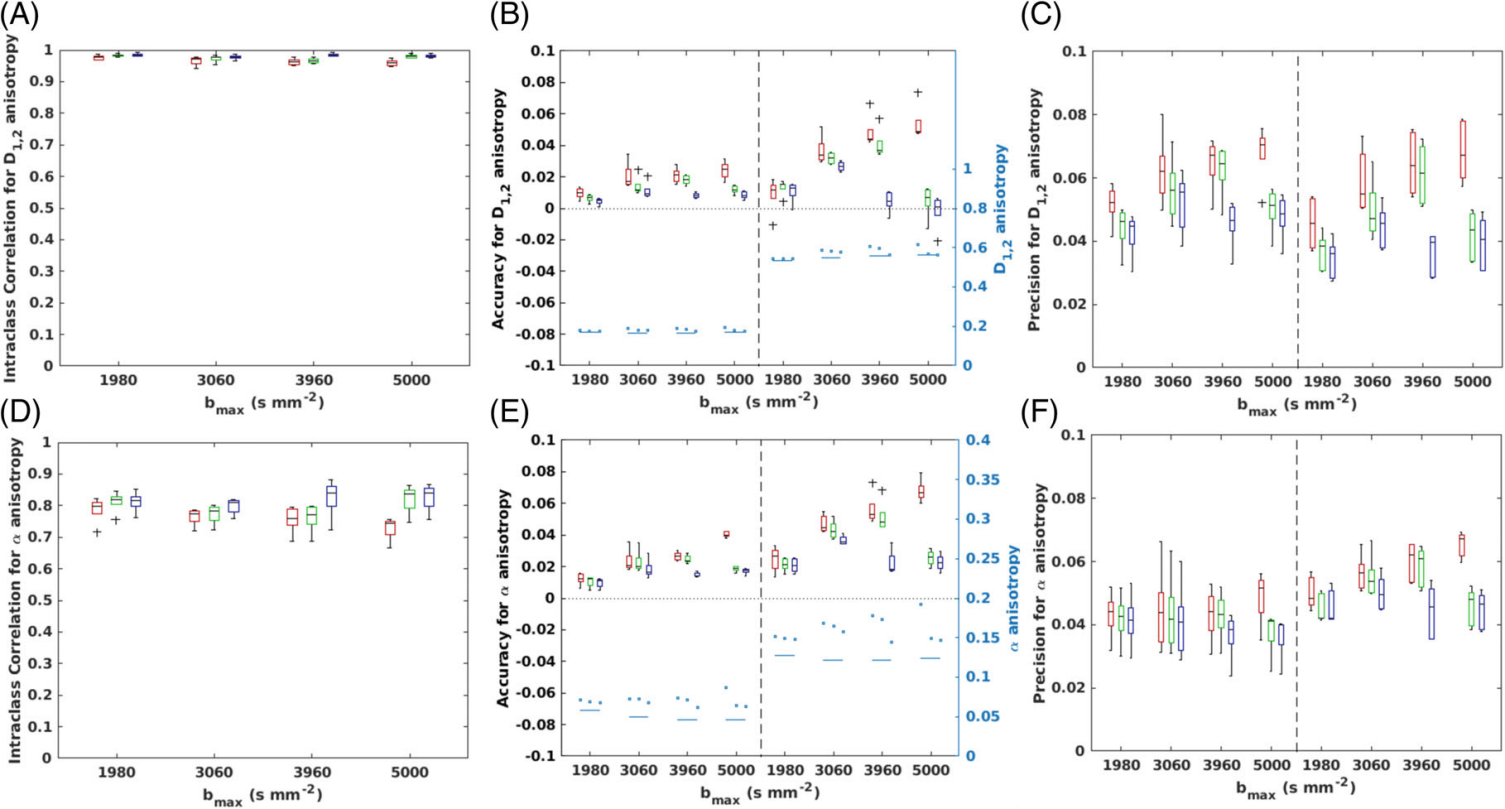
Box plots showing the reliability (intraclass correlation coefficient [ICC]), accuracy and precision (with respect to the multi‐*b*‐value reference [MbR]) of voxelwise quasi‐diffusion tensor (QDTI) measures within brain tissue. These are shown for optimal *b*‐value combinations with 2 (red), 3 (green), and 4 (blue) *b*‐value shells for $b_{\max}$ of 1980, 3060, 3960 and 5000 s mm^−2^. Plots are presented for $D_{1,2}$ anisotropy showing (A) reliability, (B) accuracy, and (C) precision, and for α anisotropy showing (D) reliability, (E) accuracy and (F) precision. Reliability was defined as excellent (ICC > 0.9), good (0.75 < ICC ≤ 0.9), moderate (0.5 < ICC ≤ 0.75) or poor (ICC ≤ 0.5). Plots of ICC show results within brain tissue. Gray matter (left) and white matter (right) results are shown in the accuracy and precision plots separated by a vertical black dashed line. The second y‐axis in the accuracy plots (colored cyan) shows the mean tissue values for the MbR (solid lines) and optimal *b*‐value combinations (circular markers).

Figures 4 and 5 show boxplots of cohort ICC values for QDTI measures within brain tissue and accuracy and precision (relative to MbR values) of QDTI measures in gray and white matter. Results for the optimal 2 *b*‐value shell combinations are shown in red. Reliability (according to ICC) was excellent for mean $D_{1,2}$ (Figure 4A) and $D_{1,2}$ anisotropy (Figure 5A) for each $b_{\max}$, although a decrease in reliability was apparent as $b_{\max}$ was increased. Good to excellent reliability was found for all $b_{\max}$ for mean α, which increased and showed less variability for higher $b_{\max}$ (Figure 4D). Reliability of α anisotropy was moderate for each $b_{\max}$ and decreased as $b_{\max}$ increased (Figure 5D).

Tissue‐specific biases were evident for QDTI estimates. In gray matter, mean $D_{1,2}$ (Figure 4B,C) and mean α (Figure 4E,F) were, in general, accurately estimated and showed consistent levels of precision across all $b_{\max}$. In white matter, mean $D_{1,2}$ was overestimated (Figure 4B,C), and mean α was underestimated (Figure 4E,F), with both effects increasing with $b_{\max}$. Increasing $b_{\max}$ also caused the precision of mean $D_{1,2}$ to decrease and the precision of mean α to increase. $D_{1,2}$ and α anisotropies were overestimated in both tissue types and became progressively less accurate and precise as $b_{\max}$ increased, however, this effect was greater in white matter than gray matter (Figure 5B,C,E,F).

**FIGURE 6:**
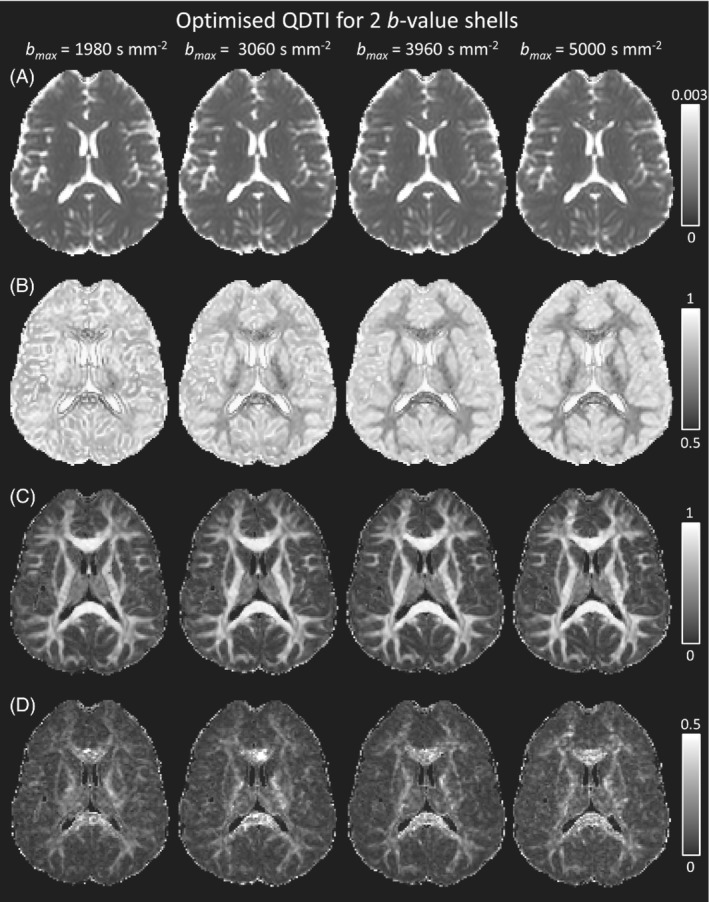
Single‐subject quasi‐diffusion tensor (QDTI) maps of (A) mean $D_{1,2}$ (in mm^2^ s^−1^), (B) mean α, (C) $D_{1,2}$ anisotropy, and (D) α anisotropy for the optimal 2 *b*‐value shell combinations for $b_{\max}$ of 1980, 3060, 3960 and 5000 s mm^−2^. Optimal acquisitions were *b* = 0, 540, 1980 s mm^−2^, *b* = 0, 900, 3060 s mm^−2^, *b* = 0, 1080, 3960 s mm^−2^, and *b* = 0, 1080, 5000 s mm^−2^. All axial images are presented using the radiological convention.

Figure 6 shows QDTI maps calculated using the optimal 2 *b*‐value shell combinations. Tissue contrast and QDTI values for the optimal combination at $b_{\max}=5000$ s mm^−2^ are consistent with previously published results for young, heathy subjects.^1^ Exceptional image quality and tissue contrast is apparent for mean $D_{1,2}$ (Figure 6A), and $D_{1,2}$ anisotropy (Figure 6B) across all $b_{\max}$ Tissue contrast for mean α increased with $b_{\max}$ (Figure 6C) and remained unchanged for mean $D_{1,2}$, and $D_{1,2}$ and α anisotropy maps. Effects of noise were apparent in α anisotropy maps for all $b_{\max}$ (Figure 6D).

#### 3.3.3 The effect of including up to 4 *b*‐value shells in the QDI optimization

**FIGURE 7:**
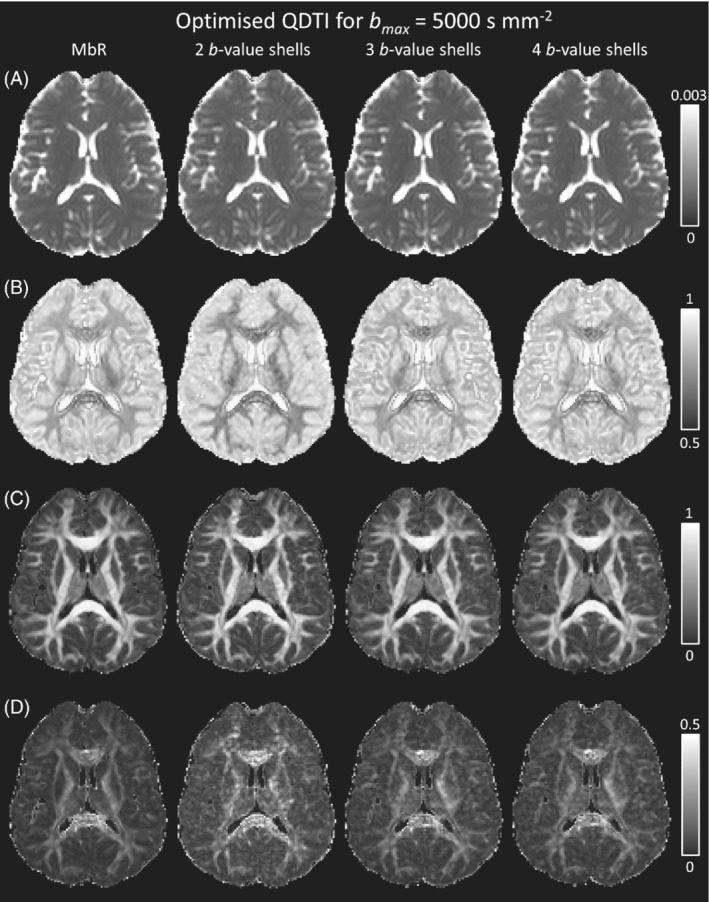
Single‐subject quasi‐diffusion tensor (QDTI) maps of (A) mean $D_{1,2}$ (in mm^2^ s^−1^), (B) mean α, (C) $D_{1,2}$ anisotropy, and (D) α anisotropy for the optimal 2, 3, and 4 *b*‐value shell combinations for $b_{\max}$ of 5000 s mm^−2^. From left to right QDTI maps are presented for the multi‐b‐value reference (MbR), optimal 2 *b*‐value shell acquisition (*b* = 0, 1080, 5000 s mm^−2^. acquisition time 3 min 12 s), optimal 3 *b*‐value shell acquisition (*b* = 0, 540, 2160, 5000 s mm^−2^, acquisition time 4 min 24 s), and optimal 4 *b*‐value shell acquisition (*b* = 0, 540, 1980, 2160, 5000 s mm^−2^, acquisition time 5 min 36 s). All axial images are presented using the radiological convention.

Figures 4 and 5 show box plots of cohort ICC values for QDTI measures within brain tissue, and the accuracy and precision of QDTI measures in gray matter and white matter for each optimal *b*‐value shell combination and $b_{\max}$ (colored red, green, and blue for 2, 3, and 4 *b*‐value shells, respectively). Increasing the number of *b*‐value shells from 2 to 4 improved the reliability of all QDTI estimates, an effect that increased the reliability of mean α estimates from good to excellent and α anisotropy estimates from moderate to good. Precision was also improved for all QDTI measures. In addition, increasing the *b*‐value shells increased the accuracy of $D_{1,2}$ and α anisotropies in both gray and white matter, but the effect was less consistent for mean $D_{1,2}$ and mean α. Nevertheless, high accuracy was achieved for gray and white matter across all QDTI measures by increasing the number of *b*‐values from 2 to 3 at $b_{\max}=5000\;$s mm^−2^ and from 3 to 4 at $b_{\max}=3960\;$s mm^−2^. Supporting Information Figure S2 shows voxelwise Bland–Altman plots of gray and white matter for $b_{\max}=5000\;$s mm^−2^ and increasing numbers of *b*‐value shells, for a single participant. The increase in accuracy and precision achieved by 3 *b*‐value shells can be clearly visualized as measurement biases are removed and 95% confidence intervals decrease. Figure 7 also shows this effect visually as all QDTI parameter maps become more similar to the MbR when the number of *b*‐value shells is increased from 2 to 3.

#### 3.3.4 The effect of $b_{\max}$ on tissue contrast for optimal *b*‐value shell combinations

Detailed results are shown in the Supporting Information section S4 and Supporting Information Figure S3. Although *t*_c_ of mean $D_{1,2}$ shows little dependence on $b_{\max}$ or number of shells, there was a trend toward small increases in α and $D_{1,2}$ anisotropy contrast with $b_{\max}$. Mean α showed a large increase in *t*_c_ with $b_{\max}$ for MbR data. Optimal *b*‐value combinations exhibited a decrease in *t*_c_ as more *b*‐value shells were included, representing *t*_c_ tending toward MbR values. *t*_c_ for mean α at $b_{\max}>3000$ s mm^−2^ was higher for 2 *b*‐value shells than 3 or 4 shells, and was the highest *t*_c_ obtained for all $b_{\max},$ including MbR maps. Supporting Information Figure S3 quantitatively confirms the visual impression in Figures 1, 6, and 7 that 2 *b*‐value shells provide the highest quality mean α maps, with greater tissue contrast at higher $b_{\max}$ (Figure 1G), and no visually apparent change in mean $D_{1,2}$, or $D_{1,2}$ and α anisotropy maps with $b_{\max}$ (Figure 1H).

## 4 DISCUSSION

We have performed a detailed analysis of the accuracy and precision of deriving QDTI measures from different *b*‐value shell combinations in comparison to those derived from a 28 *b*‐value shell reference dMRI dataset. The quasi‐diffusion model provides a robust 2‐parameter fit for $b_{\max}$ from 2000 to 5000 s mm^−2^ in individual diffusion gradient orientations, from which a tensor model provides reliable, accurate, and precise mean and anisotropy measures. In the following sections, we discuss: (1) conditions necessary for a rapid QDTI protocol that achieves the best measurement accuracy in relation to the reference; (2) compromises to accuracy and precision in using the fastest 2 *b*‐value shell acquisitions; and (3) the $b_{\max}$ dependence of QDTI measures.

Across the whole brain we observed excellent reliability (defined by ICCs) for $D_{1,2}$ measures, and lower ICCs for α, which is the lower SNR parameter, with an expected trend for ICCs to increase with inclusion of more *b*‐value shells. Each optimal *b*‐value combination included unequally spaced *b*‐values, a result consistent with similar DKI experiments.^27^, ^28^ A reproducibility study also concluded that DKI fits to *b*‐values of 1000 and 3000 s mm^−2^ showed lower coefficients of variation than to 1000 and 2000 s mm^−2^.^43^ These previous studies used maximum *b*‐values within the range commonly used for DKI, whereas we showed that a larger *b*‐value range is well‐fitted by the MLF.

Separate analyses for gray and white matter revealed significant differences. The accuracy and precision of mean $D_{1,2}$ and α measures are independent of $b_{\max}$ and the number of *b*‐value shells in gray matter, but vary significantly for white matter, where decreasing accuracy, compared to the MbR, is found with increasing $b_{\max}$ and depends on the number of *b*‐value shells. Accuracy of anisotropy in gray matter, showed similar $b_{\max}$ dependence, likely because of a proportion of white matter fibers penetrating gray matter. A systematic average decrease in mean α in white matter is consistent with our noise modeling (Figure 2) when the Rician NF becomes comparable to the tail of the decay curve at high *b*‐values. Simulation studies have shown that DKI is highly sensitive to noise and experimental design.^17^ Low SNR can lead to overestimation or underestimation of DKI parameters, which are also dependent on fitting algorithm and parameter constraints.^17^ In addition, the minimum relative error of mean K estimates is strongly dependent on $b_{\max}$, tissue type and fitting procedure.^16^ Although we have not investigated different fitting algorithms, we observed a systematic decrease in α with increased noise in all tissues, which further decreases with increasing $b_{\max}$.

Accurate estimation of α in particular, requires the deviation from Gaussian signal decay to be larger than the effects of noise. For $b_{\max}$ of 2000 s mm^−2^ the tissue signal is above the Rician NF, and on average, there is good accuracy for α across whole brain regions, irrespective of the number of *b*‐values. However, gray‐white matter tissue contrast is poor because of voxel‐by‐voxel variations in α. Higher *b*‐values lead to better image contrast, but systematic offsets in α and $D_{1,2}$ because of Rician noise, which we find can be offset by inclusion of additional *b*‐value shells. For $b_{\max}$ of 5000 s mm^−2^ our optimization suggests the best accuracy and precision is achieved with b={0,540,2160,5000} s mm^−2^, or with 4 *b*‐values of b={0,540,1620,2160,3960} s mm^−2^ for lower $b_{\max}$.

The most rapid QDTI acquisition requires only 2 *b*‐value shells. Although decreased accuracy is observed with increasing $b_{\max}$, there is greatly improved image quality and contrast. Therefore, we suggest that 2 *b*‐value shells with a $b_{\max}\geq 3960$ s mm^−2^ provides stable measurements and high contrast images. It remains to be determined whether optimization of high‐angular resolution data would identify the same optimal *b*‐value combinations and would provide averaging that improves the accuracy and precision of QDTI measure estimates in the same way as increasing the number of *b*‐value shells. An acquisition protocol including b={0,1100,4000} s mm^−2^ represents a good candidate for future high resolution QDI experiments as higher *b*‐values and greater numbers of diffusion gradient directions are reported to improve white matter fiber tractography.^44^, ^45^, ^46^, ^47^, ^48^ For this analysis, rapid acquisitions were optimized relative to MbR data comprising all *b*‐values up to a given $b_{\max}$ This ensured that the signal‐to‐noise of the MbR was maximized, but may have introduced bias, as the MbR comprised different numbers of *b*‐values.

To ensure high SNR we acquired dMRI data with 2 signal averages. Acquisition time reductions could include no data averaging to obtain 2 *b*‐value shell QDTI full brain coverage in 2 min, if lower SNR is acceptable,^1^ or reducing the number of b=0 s mm^−2^ acquisitions. Other applicable methods include compressed sense, multi‐band imaging, and deep‐learning based image reconstruction from undersampled k‐space data.^49^, ^50^ Additionally, neural network techniques show promise for improving the SNR of rapidly acquired QDI maps.^51^ Fast DKI protocols have also been developed, which acquire 13 or 19 dMR images (termed 1‐3‐9 and 1‐9‐9 protocols),^52^, ^53^, ^54^ but require additional assumptions for calculating the full kurtosis tensor.^54^ Currently, we use a symmetric second‐order α tensor representation, compared to DKI, which includes a fourth‐order K tensor representation. The simpler representation used by QDTI could generate orientational dependence of anisotropy and is a potential limitation of the technique. Further work is needed to assess high‐angular resolution QDTI for detailed characterization of complex microstructural tissue geometries such as crossing fibers, and to develop QDTI using concepts from anisotropic fractional diffusion imaging^55^ to provide an improved representation of the α tensor.

Our MbR data shows significant changes in α and $D_{1,2}$ with $b_{\max}$, hence our optimization was to $b_{\max}$ specific reference values. The <2% reductions in gray matter α over the range $b_{\max}$ 2000‐5000 s mm^−2^ are within the expected range for a Rician noise offset, but reductions of ≈7% in white matter are larger than expected (≈4%). In addition, we predominantly observe a small reduction in $D_{1,2}$, the largest effect for radial white matter (≈5%) with only axial $D_{1,2}$ showing an increase (≈1%) as expected for Rician noise. Therefore, there appear to be systematic changes in QDTI measures that are not explained by the effects of Rician noise, but may indicate greater sensitivity to tissue microstructure at higher $b_{\max}$. These comparatively small changes in QDTI measures up to $b_{\max}$ 5000 s mm^−2^ contrast to observations of 10%‐20% variability in both $D_{\mathrm{app}}$ and K for DKI with $1500\leq b_{\max}\leq 3000$ s mm^−2^.^56^, ^57^ In normal brain, $D_{\mathrm{app}}$ and K decreased with increasing $b_{\max}$, with $D_{\mathrm{app}}$ always higher than reference values from a multi‐*b*‐value acquisition, whereas variation in K ranged from below to above reference values.^56^ Therefore, QDI appears to provide a more stable parameterization of the dMRI signal decay than DKI.

The functional form of QDI (Equation [2]) means that α describes the inverse power law of the dMRI signal at high *b*‐value, and our results in gray and white matter are consistent with experimental results for power laws relating to spherically averaged dMRI signal for $b_{\max}$ of 6000 s mm^−2^,^58^ and higher.^59^ It remains to be determined whether our mean α tends to 0.5 for ultrahigh $b_{\max}$ as would be predicted by Veraart et al,^59^ however, were these 2 limits to coincide it would be evidence of a deep connection between the approaches, which may be useful in further theoretical and analytical developments.

Further work is required to understand how the observed QDI $b_{\max}$ dependence relates to tissue microstructure by focusing measurements within specific white matter regions of known structure, and acquiring data to accurately assess the background noise, such as by acquiring complex data to avoid Rician noise bias.^60^ Our preliminary results suggest high $b_{\max}$ provide greatest contrast in α, and so greatest potential for detecting pathological change. Although our optimization is over healthy brain, we believe our protocols would be suitable for studies of non‐Gaussian diffusion in aging and neurodegenerative diseases, but further work is needed to assess their optimization for lesional tissue such as brain tumors or stroke.

## 5 CONCLUSION

We have shown that QDI provides robust parameterization of non‐Gaussian diffusion signal decay in clinically feasible imaging times with high reliability, accuracy, and precision of QDTI measures without the need for noise correction. This extends parameterization of the dMRI signal decay to higher *b*‐values than routinely acquired for DKI and takes advantage of increased sensitivity to microstructural properties. QDI can be used to robustly fit dMRI signal decay in single diffusion gradient directions without restrictions of cumulant expansions. For optimal accuracy, precision, and image contrast, a 3 *b*‐value shell acquisition with $b_{\max}$ of 5000 s mm^−2^ is preferred (acquisition time 156 s for 1 average with 8 b=0 s mm^−2^ images and TR = 6 s). $b_{\max}$ of 4000‐5000 s mm^−2^ provides the highest image contrast for α, and, if reduced accuracy and precision is acceptable, a 2 *b*‐value shell acquisition will enable a shorter acquisition time of 120 s. Further work is required to investigate the systematic changes in $D_{1,2}$ and α with $b_{\max}$ within specific white matter regions while including precise compensation for noise.

## CONFLICTS OF INTEREST

The QDI technique is covered by patent application GB1909982.9 published as WO 2021/005363 on January 14, 2021 (inventors: Dr. T.R. Barrick, Prof. F.A. Howe, Dr. M.G. Hall, Dr. C. Ingo, Prof. R.L. Magin).

## Supporting information


**FIGURE S1** Signal attenuation and fitted QDI signal decay curves for representative gray matter and white matter voxels shown in each diffusion gradient direction. The anatomic location of the gray (red arrow) and white matter (blue arrow) voxels are shown on axial slices of mean $D_{1,2}$ and mean α maps.
**FIGURE S2** Voxelwise Bland–Altman plots showing quantitative differences between QDTI measures and the MbR for a $b_{\max}$ of 5000 s mm^−2^ for optimal 2, 3, and 4 *b*‐value shell combinations. Plots are shown for (A) mean $D_{1,2}$, (B) mean α, (C) $D_{1,2}$ anisotropy, and (D) α anisotropy for voxels from a single representative subject. Solid horizontal lines indicate the mean difference (accuracy) and dashed horizontal lines indicate the 95% lower and upper confidence limits (precision). Gray matter voxels = red, white matter voxels = blue.
**FIGURE S3** Plot showing the cohort average tissue contrast (*t*_c_) between gray and white matter for QDTI measures with respect to $b_{\max}$ for MbR (solid), and 2 (dotted), 3 (short dashes) and 4 *b*‐value shells (long dashes). Mean $D_{1,2}$ = blue, mean α = green, $D_{1,2}$ anisotropy = gray and α anisotropy = orange
