# Supplementary material for: Optimization of quasi‐diffusion magnetic resonance imaging for quantitative accuracy and time‐efficient acquisition
Source: Magn Reson Med. 2022 Aug 31;88(6):2532–47. doi: 10.1002/mrm.29420 (PMC9804504; doi:10.1002/mrm.29420)
Supplement: Supplementary file 1 — FIGURE S1 Signal attenuation and fitted QDI signal decay curves for representative gray matter and white matter voxels shown in each diffusion gradient direction. The anatomic location of the gray (red arrow) and white matter (blue arrow) voxels are shown on axial slices of mean D1,2 and mean α maps. FIGURE S2 Voxelwise Bland–Altman plots showing quantitative differences between QDTI measures and the MbR for a bmax of 5000 s mm−2 for optimal 2, 3, and 4 b‐value shell combinations. Plots are shown for (A) mean D1,2, (B) mean α, (C) D1,2 anisotropy, and (D) α anisotropy for voxels from a single representative subject. Solid horizontal lines indicate the mean difference (accuracy) and dashed horizontal lines indicate the 95% lower and upper confidence limits (precision). Gray matter voxels = red, white matter voxels = blue. FIGURE S3 Plot showing the cohort average tissue contrast (t c) between gray and white matter for QDTI measures with respect to bmax for MbR (solid), and 2 (dotted), 3 (short dashes) and 4 b‐value shells (long dashes). Mean D1,2 = blue, mean α = green, D1,2 anisotropy = gray and α anisotropy = orange [file MRM-88-2532-s001.pdf]

Supporting information:

Optimization of Quasi-Diffusion Magnetic Resonance Imaging (QDI) for quantitative accuracy and time-efficient acquisition

Catherine A. Spilling[1,2*], Franklyn A. Howe[1], Thomas R. Barrick[1]

1. Neurosciences Research Section, Molecular and Clinical Sciences Research Institute, St George's, University of London, London, UK
2. Centre for Affective Disorders, Department of Psychological Medicine, Division of Academic Psychiatry, Institute of Psychiatry, Psychology & Neuroscience, King's College London, London, UK

***Corresponding author details:**

Neurosciences Research Section

Molecular and Clinical Sciences Research Institute

St George's, University of London

Cranmer Terrace

London, SW17 0RE

Email: cspillin@sgul.ac.uk (CAS)

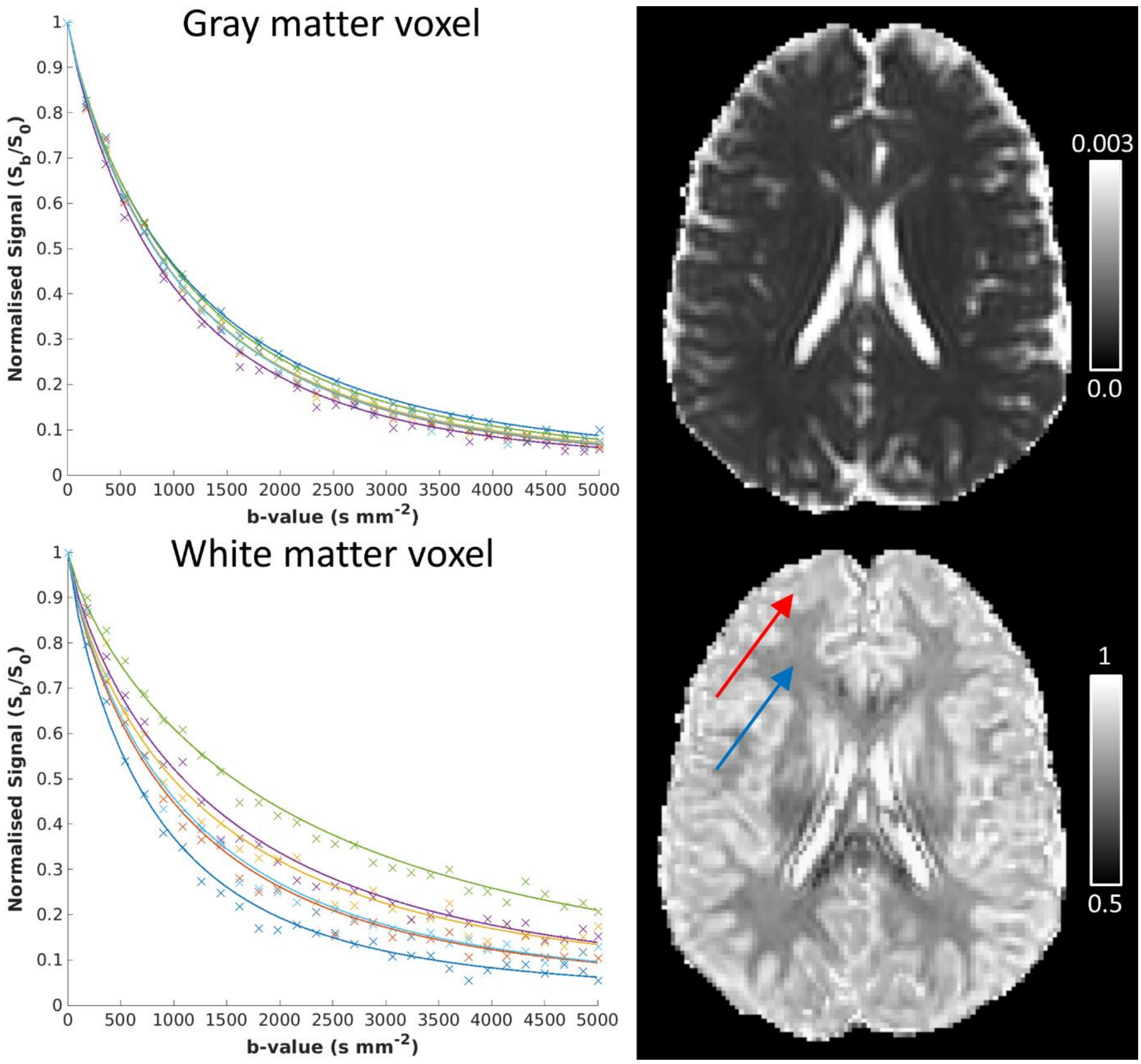

Supporting Information Figure S1: Signal attenuation and fitted QDI signal decay curves for representative gray matter and white matter voxels shown in each diffusion gradient direction. The anatomical location of the gray (red arrow) and white matter (blue arrow) voxels are shown on axial slices of mean $D_{1,2}$ and mean α maps.

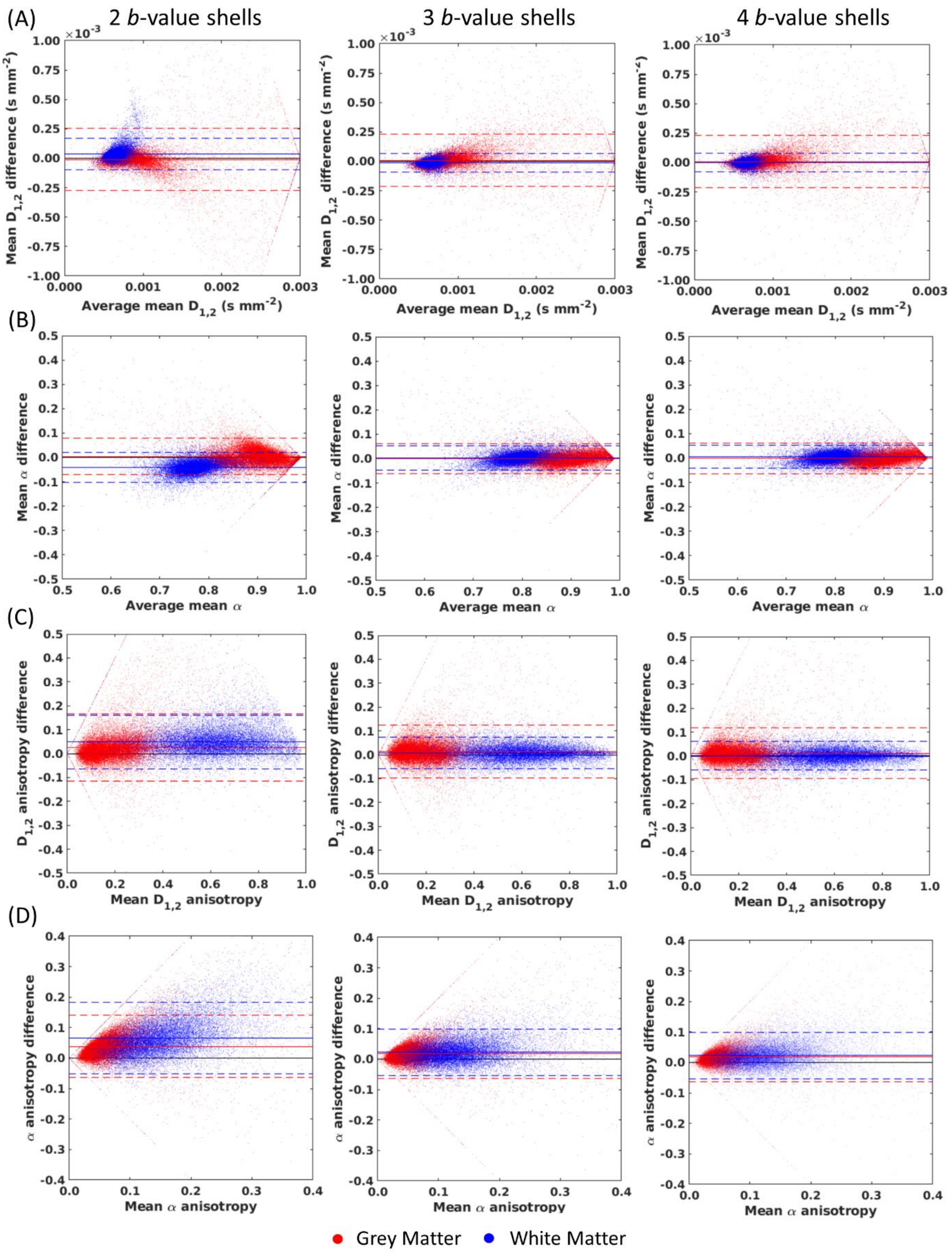

Supporting Information Figure S2: Voxelwise Bland-Altman plots showing quantitative differences between QDTI measures and the MbR for a b_{max} of 5000 s mm^{-2} for optimal 2, 3 and 4 b-value shell combinations. Plots are shown for (A) mean $D_{1,2}$, (B) mean α, (C) $D_{1,2}$ anisotropy, and (D) α anisotropy for voxels from a single representative subject. Solid horizontal lines indicate the mean difference

(accuracy) and dashed horizontal lines indicate the 95% lower and upper confidence limits (precision). Gray matter voxels = red, white matter voxels = blue.

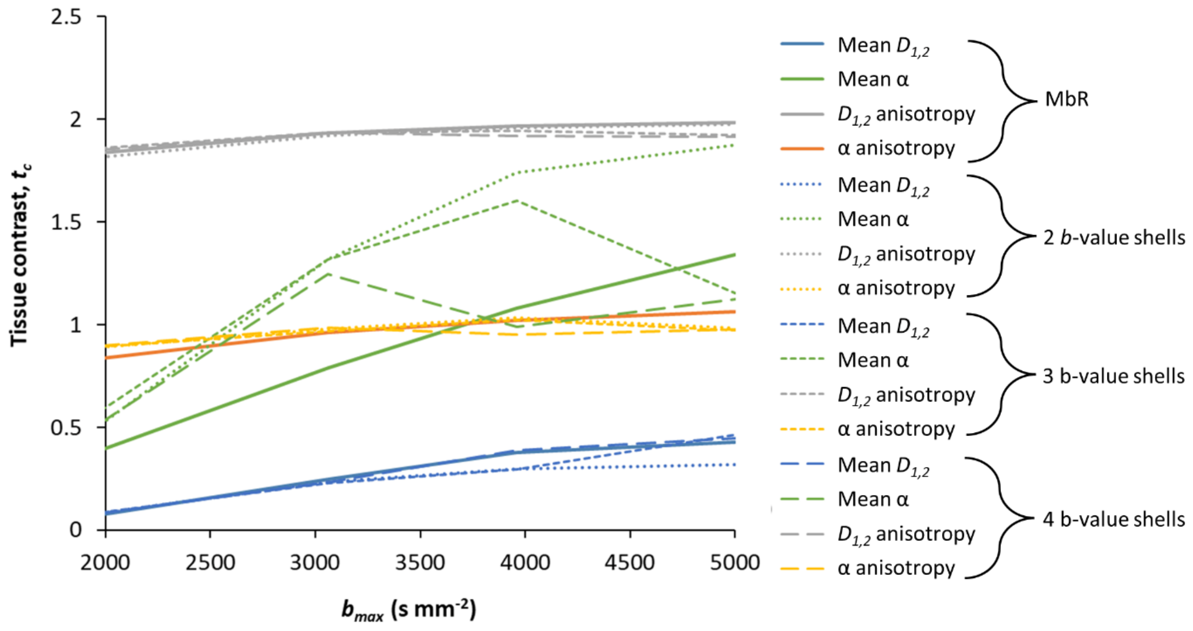

Supporting Information Figure S3: Plot showing the cohort average tissue contrast (t_c) between gray and white matter for QDTI measures with respect to b_{max} for MbR (solid), and 2 (dotted), 3 (short dashes) and 4 b-value shells (long dashes). Mean $D_{1,2}$ = blue, mean α = green, $D_{1,2}$ anisotropy = gray and α anisotropy = orange.

S1. Region of interest segmentation of gray and white matter and brain tissue

T1-weighted images were segmented into gray matter, white matter and cerebrospinal fluid (CSF) using New Segment (SPM version 12, https://www.fil.ion.ucl.ac.uk/spm/) [1]. The b = 0 s mm^{-2} were co-registered to the T1-weighted images using 'epi_reg' in FSL (version 5.0.11, https://fsl.fmrib.ox.ac.uk/fsl/fslwiki/).[2] These transformations were inverted and applied to the gray matter, white matter, and CSF tissue segmentations to align them with the dMRI data. To minimize partial volume effects, low tissue probabilities (< 0.95) were excluded. Gray matter, white matter and whole brain tissue (gray and white matter) regions of interest (ROIs) were constructed.

S2. Investigation of the effect of Rician noise on quasi-diffusion model fitting

Background noise was estimated from ventricular cerebrospinal fluid (CSF) regions at the highest acquired b-value (b = 5000 s mm^{-2}) for which we assumed there was no tissue signal. A central axial slice of the b = 0 s mm^{-2} images of each subject was manually thresholded in ImageJ (https://imagej.nih.gov/ij/)[3] at a sufficiently high signal level to give CSF regions of interest (ROIs) of typically 350 voxels that had a boundary that was not immediately adjacent to brain tissue. The ROIs were used as a CSF mask which was applied to the b = 5000 s mm^{-2} dMRIs along each diffusion gradient direction, and the average noise level was determined. We then estimated the standard deviation of Gaussian noise, σ, from the relationship that the average Rician noise, μ_R, is given by[4]

$$\mu_R = \sigma\sqrt{\pi/2}. \qquad \text{[S.1]}$$

The effect of noise on QDI parameter estimation was investigated by creating noise free signal decay curves with $D_{1,2}$ and α parameters appropriate for CSF, gray matter and callosal white matter to which Gaussian noise was added. Noise was added separately to the real (the MLF signal decay curve) and imaginary (set equal to zero) signals at each b-value that was obtained in our dMRI acquisition (see Section 2.2) before calculating the magnitude signal decay curve to simulate a signal with a Rician noise distribution.[5] $D_{1,2}$ and α were then estimated by application of our quasi-diffusion fitting algorithm (see Section 2.3.2) to the simulated noisy dMRI data across a range of b-values with different maximum b_{max} in the range $1980 \leq b_{max} \leq 5000$ s mm^{-2} in steps of approximately 500 s mm^{-2}. Noise simulations were performed 1000 times for each noise level. The noise levels were representative of potential noise within the normalized signal decay curves (S_b/S_0) in CSF ($\sigma = \{0.002, 0.004, 0.006, 0.008, 0.01\}$) and gray and white matter ($\sigma = \{0.01, 0.02, 0.03, 0.04, 0.05\}$). The means and standard deviations of estimated $D_{1,2}$ and α values from the simulated noisy decay curves were then calculated for each noise level.

S3. Effect of Rician noise on quasi-diffusion model fitting

Histogram analysis of the b = 5000 s mm^{-2} CSF ROIs revealed a skewed distribution indicative of Rician noise with an average value (in arbitrary units) of 15.5 (range 14.4 to 16.8). After signal normalization based on the typical b = 0 s mm^{-2} signal intensity in the central slice of CSF, gray matter and white matter regions the Gaussian noise standard deviation was calculated as σ = 0.007 in CSF, σ = 0.022 in gray matter, and σ = 0.031 in white matter. These σ values were used for inference of the effect of Rician noise within CSF, gray matter and white matter. Figure 2 (top row) shows noise free quasi-diffusion signal decay curves for the expected $D_{1,2}$ and α within CSF ($D_{1,2} = 3.0 \times 10^{-3}$ mm^2 s^{-1} and $\alpha = 1$), and for average $D_{1,2}$ and α values obtained from manually defined ROIs in the gray matter of the anterior cingulate cortex (mean $D_{1,2} = 0.8 \times 10^{-3}$ mm^2 s^{-1} and mean $\alpha = 0.92$), and white matter of the corpus callosum (axial $D_{1,2} = 1.9 \times 10^{-3}$ mm^2 s^{-1} and axial $\alpha = 0.91$; radial $D_{1,2} = 0.4 \times 10^{-3}$ mm^{2}s^{-1} and radial $\alpha = 0.76$). The dotted line indicates the Rician noise floor for the normalized signal decay curve for each tissue type. Gaussian noise within the range $0.002 \leq \sigma \leq 0.01$ for CSF, and within $0.01 \leq \sigma \leq 0.05$ for gray and white matter, was then applied to the tissue quasi-diffusion decay curves and estimates of $D_{1,2}$ and α were calculated from the noisy data for b-values in the range $1980 \leq b_{max} \leq 5000$ s mm^{-2} as shown in Figure 2 (middle and bottom rows).

S4. Tissue Contrast

We have previously defined a tissue contrast parameter, t_c [6], that we have used as a measure of image clarity in delineation of gray and white matter,

$$t_c = \frac{|\mu_{white} - \mu_{grey}|}{\sqrt{\sigma^2_{white} + \sigma^2_{grey}}}, \qquad [S.2]$$

where μ and σ are the mean and standard deviation of a measure determined across segmented white and gray matter of the whole brain. Note, the value of σ will comprise biological variability as well as noise variance. Higher t_c indicates greater tissue contrast and clearer separation of gray and white matter values. Supporting Information Figure S3 shows tissue contrast between gray and white matter for each QDTI measure as a function of b_{max} for the MbR data, and for the optimal 2, 3 and 4 b-value shell combinations. There is a trend for modest increases in contrast with b_{max} for $D_{1,2}$ and α anisotropy, which is independent of the number of b-shells used in the acquisition. Contrast for mean $D_{1,2}$ remains constant and for mean α shows large increases of a factor of approximately 4, as b_{max} increases from 2000 to 5000 s mm^{-2}. Contrast for mean α is highly dependent on the number of b-shells. For the optimal 3 and 4 b-value shell combinations the tissue contrast initially increases with b_{max} before reducing to converge to the MbR value at b_{max} 5000 s mm^{-2}. The optimal 2-shell acquisition shows the highest tissue contrast in α for all b_{max}, and shows a strong increase with b_{max} before stabilising between b-

values of 4000 to 5000 s mm^{-2}. This is reflected in increased image quality of gray and white matter delineation in Figure 1g.

References

1. Ashburner J, Friston KJ. Unified segmentation. *NeuroImage*. 2005;26(3):839-851. doi:10.1016/j.neuroimage.2005.02.018

2. Greve DN, Fischl B. Accurate and robust brain image alignment using boundary-based registration. *NeuroImage*. 2009;48(1):63-72. doi:10.1016/j.neuroimage.2009.06.060

3. Rasband WS. *ImageJ*. Bethesda, Maryland, USA: U. S. National Institutes of Health; 1997-2018. https://imagej.nih.gov/ij/.

4. Gudbjartsson, H, Patz, S. The Rician distribution of noisy MRI data. *Magn Reson Med*. 2008; 34(6): 910-914. doi: 10.1002/mrm.1910340618

5. Sijbers J, den Dekker AJ, Scheunders P, Van Dyck D. Maximum-likelihood estimation of Rician distribution parameters. *IEEE Trans Med Imaging*. 1998;17(3):357-361. doi:10.1109/42.712125

6. Barrick TR, Spilling CA, Ingo C, et al. Quasi-diffusion magnetic resonance imaging (QDI): A fast, high b-value diffusion imaging technique. *NeuroImage*. 2020;211:116606. doi:10.1016/j.neuroimage.2020.116606
